# Anti-Inflammatory Effect of Liverwort (*Marchantia polymorpha* L.) and Racomitrium Moss (*Racomitrium canescens* (Hedw.) Brid.) Growing in Korea

**DOI:** 10.3390/plants10102075

**Published:** 2021-09-30

**Authors:** So-Yeon Kim, Minji Hong, Tae-Hee Kim, Ki Yeon Lee, Se Jin Park, Sun Hee Hong, Kandhasamy Sowndhararajan, Songmun Kim

**Affiliations:** 1 School of Natural Resource and Environmental Science, Kangwon National University, Chuncheon 24341, Korea; ykims95@kangwon.ac.kr (S.-Y.K.); alswl0356@kangwon.ac.kr (M.H.); hanmi230@naver.com (T.-H.K.); lky6520@korea.kr (K.Y.L.); sejinpark@kangwon.ac.kr (S.J.P.); 2 Agriproduct Processing Experiment Station, Gangwon-do Agriculture Research and Experiment Services, Chuncheon 24203, Korea; 3 Department of Plant Life and Environmental Science, Hankyong National University, Ansung 17579, Korea; shhong@hknu.ac.kr; 4 Department of Botany, Kongunadu Arts and Science College, Coimbatore 641029, India

**Keywords:** liverwort, *Marchantia polymorpha*, *Racomitrium canescens*, anti-inflammatory, nitric oxide, HaCaT cell

## Abstract

Bryophytes contain a variety of bioactive metabolites, but studies about the anti-inflammatory effect of bryophytes are meager. Therefore, the present study aimed to compare the anti-inflammatory effect of methanol extract of *Marchantia polymorpha* L. (liverwort) and *Racomitrium canescens* (Racomitrium moss) in lipopolysaccharide (LPS)-induced HaCaT cells. To evaluate the anti-inflammatory effect of liverwort and Racomitrium moss, the levels of nitric oxide (NO) production and the mRNA expression of inducible nitric oxide synthase (iNOS), cyclooxygenase-2 (COX-2) and tumor necrosis factor-α (TNF-α), and interleukin (IL)-6 and IL-1β in LPS-induced HaCaT cells were measured. The methanol extract of liverwort and Racomitrium moss significantly decreased LPS-induced NO production in HaCaT cells. When compared with Racomitrium moss extract, pre-treatment with methanol extract of liverwort markedly inhibited the expression of iNOS, COX-2, IL-6, and IL-1β at the concentration of 100 µg/mL with the exception of TNF-α. Further, liverwort extract markedly attenuated the production of TNF-α, IL-6, and IL-1β in the culture medium. In addition, ethyl acetate and butanol fractions obtained from the methanol extract of liverwort showed remarkable inhibitory activity against the production of NO in LPS-stimulated HaCaT cells. The LC-MS data revealed the presence of bisbibenzyl types of bioactive components in the methanol extract of liverwort. These data demonstrate that liverwort extract exhibits effective inhibitory activity against the production of inflammatory mediators in LPS-induced HaCaT cells and may be useful for the treatment of inflammation-mediated diseases.

## 1. Introduction

The human epidermis, mainly comprised of keratinocytes (about 95%), is a principal portion of the skin’s immune system. Keratinocytes provide the first line of defense against various external harmful agents, such as microorganisms and toxic chemicals [1,2]. In the inflamed skin, keratinocytes play an important role in the structural integrity of skin and the inflammatory responses by producing various pro-inflammatory cytokines such as interleukin-6 (IL-6), IL-1β, tumor necrosis factor-α (TNF-α), inducible nitric oxide synthase (iNOS) derived nitric oxide (NO), and cyclooxygenase-2 (COX-2) prostaglandins [3]. Cytokines are the most important contributors in the regulation of the immune system. Although cytokines and other mediators contribute to normal homeostatic mechanisms in the skin, overproduction of pro-inflammatory mediators may lead to various inflammatory diseases [4]. Hence, the suppression of pro-inflammatory mediators may play a key role in the treatment of inflammation-mediated skin diseases.

Many synthetic drugs have shown an appreciable anti-inflammatory effect but they have certain side effects, including gastric bleeding and ulceration [5]. Therefore, there is a growing trend to develop safer natural products for the treatment of inflammation-mediated diseases. Lipopolysaccharides (LPS), the major component of the Gram-negative bacterial cell wall, are one of the most powerful stimulators of the production of pro-inflammatory cytokines [6]. The HaCaT cell line is routinely employed to confirm the effects of drugs on epidermal inflammation. Hence, the inhibition of pro-inflammatory mediator’s production in LPS-stimulated HaCaT cells is a potential way to screen the anti-inflammatory effect of extracts/compounds.

In the plant kingdom, bryophytes are the second most diverse group of terrestrial plants after angiosperms with about 25,000 species found throughout the world. Bryophytes are classified into mosses, liverworts, and hornworts [7]. They contain a variety of biologically active components such as fatty acids, terpenoids, flavonoids, and polyphenols [7,8,9]. In traditional systems of medicine, bryophyte plants have been used to cure various ailments in India, China, and North America, including burns, external wounds, snake bites, pulmonary tuberculosis, cardiovascular diseases, bone fractures, hepatic disorders, and skin diseases [10].

**Figure 1:**
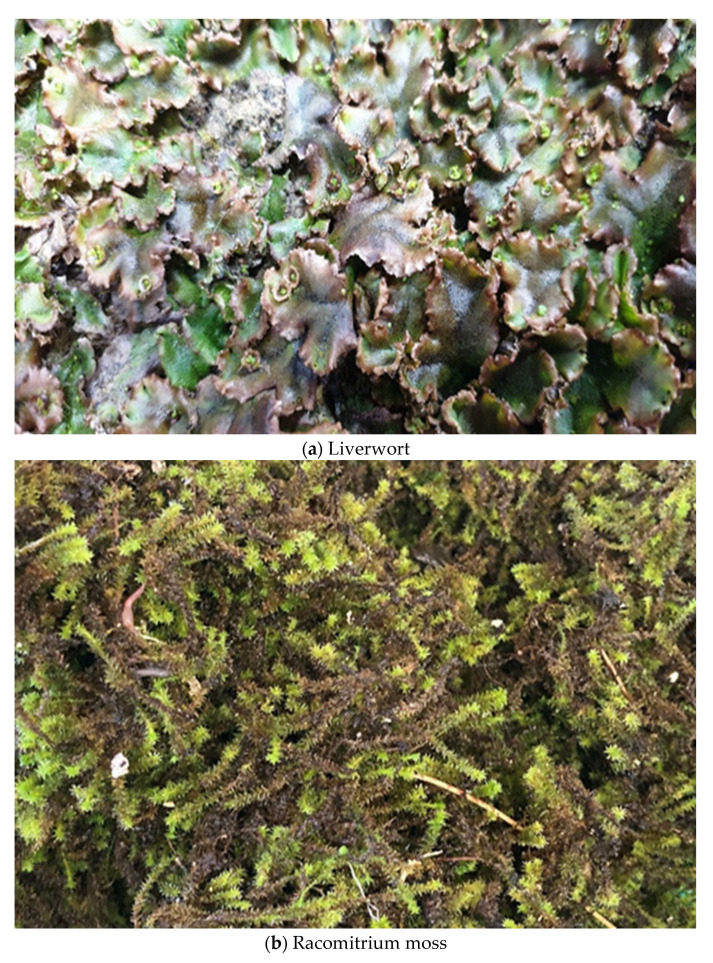
Morphology of liverwort and Racomitrium moss.

Mosses are the largest group of bryophytes, playing a significant role in the ecosystem of terrestrial biodiversity. Traditionally, mosses have been used to treat wounds, burns, and other diseases. In these, the genus *Racomitrium* (Grimmiaceae) is an important component of various terrestrial ecosystems [11]. *Racomitrium canescens* (Hedw.) Brid. (Racomitrium moss) is widely distributed from the northern temperate to arctic zones (Figure 1). However, only a few studies have been conducted on this genus, especially from a taxonomic point of view [11]. Liverworts are the second important group of bryophytes, containing about 6000 species, and are considered to be the oldest aquatic-terrestrial plants [12,13]. Liverworts mainly contain volatile components (terpenoids) in addition to a wide variety of other bioactive components. In particular, some terpenoid compounds such as the pinguisane group of sesquiterpenoids and the sacculatane group of diterpenoids are not identified in other plant species [14]. *Marchantia* is one of the important genera in the family of Marchantiaceae. The common liverwort *Marchantia polymorpha* L. (liverwort) is mainly found in temperate regions (Figure 1) [15]. This plant has been traditionally used to treat boils, fractures, poisonous snakebites, abscesses, wounds, and hepatic disorders. Liverwort has also been used as a diuretic agent in European countries [10,16,17]. The extracts and compounds isolated from liverwort exhibited various biological activities such as antimicrobial [17,18], antioxidant [19], anti-inflammatory [20] activities, and cytotoxic potential against cancer cell lines [21]. However, there has been no study in relation to the anti-inflammatory potential of liverwort and Racomitrium moss in LPS-induced HaCaT cells.

Based on the highly acclaimed properties of liverwort and Racomitrium moss, we investigated the anti-inflammatory effects of methanol extract of liverwort and Racomitrium moss by measuring its ability to inhibit NO production and mRNA expression of iNOS, COX-2, TNF-α, IL-6, and IL-1β in LPS-stimulated HaCaT cells.

## 2. Results and Discussion

### 2.1. The Effect of Extract on the Viability of HaCaT Cells

**Figure 2:**
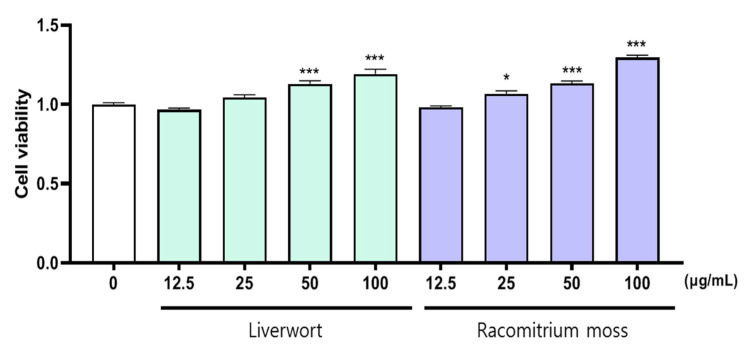
The effect of methanol extract of liverwort and Racomitrium moss on the cell viability of HaCaT cells. Values are mean of three replicate determinations (*n* = 3) ± standard deviation. * *p* < 0.05, *** *p* < 0.001 vs. 1% MeOH alone.

Keratinocytes, a major part of epidermal cells, play an important role in the pathogenesis of inflammatory skin lesions by producing pro-inflammatory mediators [22,23]. First, we determined the viability of HaCaT cells in the presence of methanol extracts of liverwort and Racomitrium moss for 24 h to evaluate their possible cytotoxic effect. For this purpose, the HaCaT cells were incubated with different concentrations of methanol extracts of liverwort and Racomitrium moss. After the treatment, the survival of HaCaT cells was not significantly affected by 24 h incubation with up to 100 µg/mL concentration of both the extracts (cell viability > 90%) (Figure 2). Therefore, non-toxic concentrations of liverwort and Racomitrium moss extracts up to 100 µg/mL were used for further experiments.

### 2.2. Inhibition of Nitric Oxide Production in LPS-Stimulated HaCaT Cells

**Figure 3:**
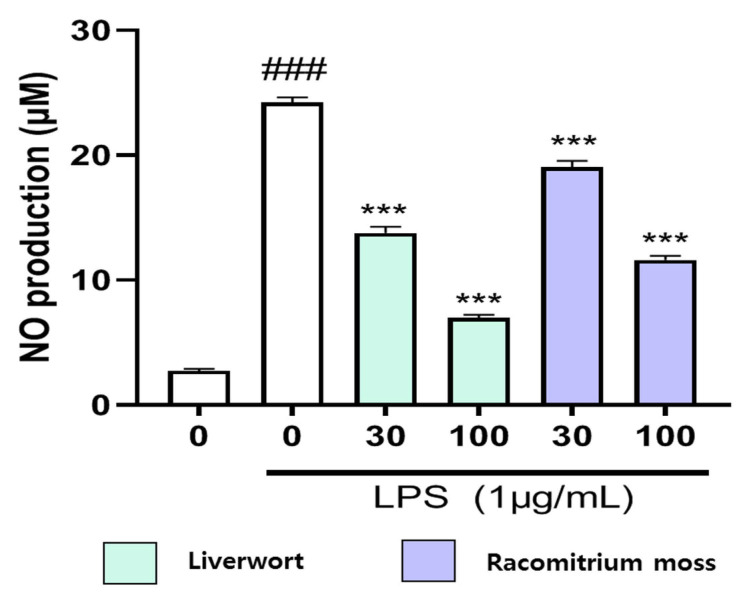
Inhibitory effect of methanol extract of liverwort and Racomitrium moss on NO production by LPS-stimulated HaCaT cells. Values are mean of three replicate determinations (*n* = 3) ± standard deviation. ### *p* < 0.001 vs. 1% MeOH alone, *** *p* < 0.001 vs. LPS alone.

It is well established that LPS can lead to the release of various pro-inflammatory cytokines, including adhesion molecules such as nitric oxide (NO) [24]. Under normal physiological conditions, NO regulates many biological functions such as host defense, platelet aggregation, vasoregulation, and neurotransmission. However, excessive production of NO and other inflammatory mediators is linked with the development of many diseases [25]. Hence, we determined the inhibitory effect of the methanol extract of liverwort and Racomitrium moss on NO production in LPS-induced HaCaT cells (Figure 3).

To accomplish this experiment, HaCaT cells were activated by LPS, and the production of NO was measured as nitrite concentration in the cell culture supernatant. The LPS treatment (1 µg/mL) significantly increased NO production by HaCaT cells by accumulating a higher level of nitrite (24.24 µM). To determine the effect of liverwort and Racomitrium moss extracts on NO production, cells were simultaneously treated with 1 µg/mL LPS and two different concentrations of extracts (30 and 100 µg/mL). When compared to the untreated control, the cells pre-treated with methanol extracts of liverwort and Racomitrium moss significantly (*p* < 0.001) decreased the production of NO in the medium to 6.99 and 11.61 µM, respectively, at the concentration of 100 µg/mL (Figure 3). The inhibitory effect of liverwort and Racomitrium moss extracts on NO production was not owing to the damage of cells (viability > 90%) as measured in the MTT cell viability assay. There has been no study on the inhibitory effect of moss or liverworts on NO production in HaCaT cells. However, these results were comparable with those previously obtained by other researchers on RAW 264.7 cells. In LPS-induced RAW 264.7 cells, compounds isolated from liverworts such as *Mastigophora diclados* [26], *Porella densifolia* [27], *Lepidozia reptans* [28], and *Jamesoniella autumnalis* [29] showed a potent inhibitory effect on the production of NO.

### 2.3. The Effect of Extracts on mRNA Expression of iNOS, COX-2, TNF-α, IL-6, and IL-1β

**Figure 4:**
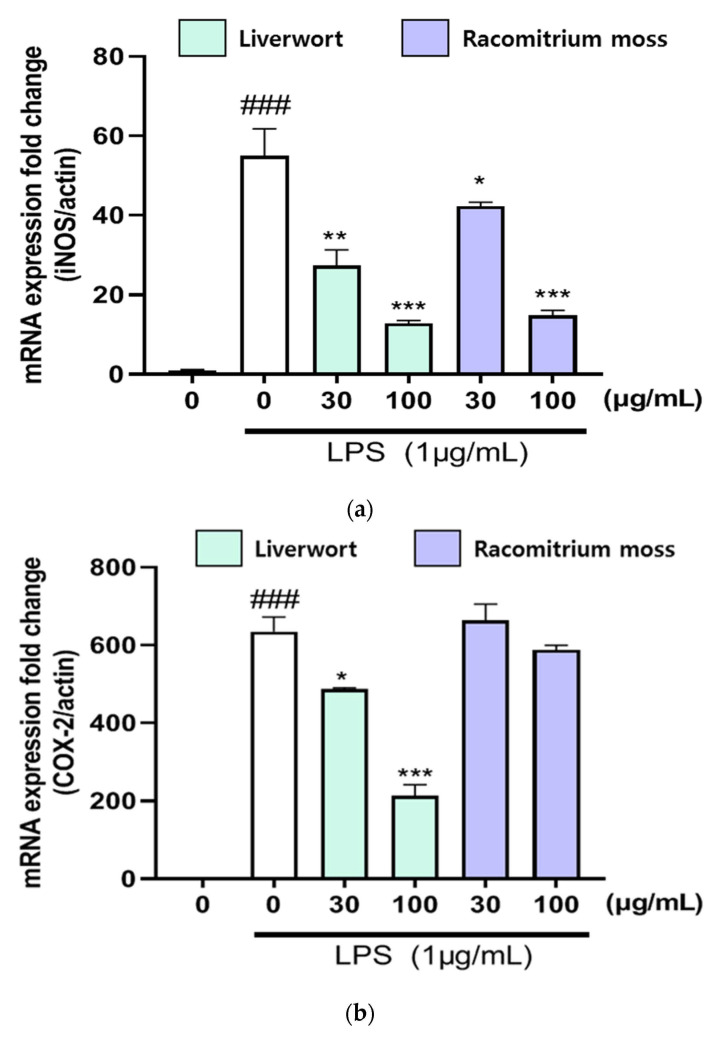
The effect of methanol extract of liverwort and Racomitrium moss on mRNA expressions of iNOS and COX-2 in LPS-stimulated HaCaT cells. (**a**) mRNA expression of iNOS; (**b**) mRNA expression of COX-2. Values are mean of three replicate determinations (*n* = 3) ± standard deviation. ### *p* < 0.001 vs. 1% MeOH alone, * *p* < 0.05, ** *p* < 0.01, *** *p* < 0.001 vs. LPS alone.

**Figure 5:**
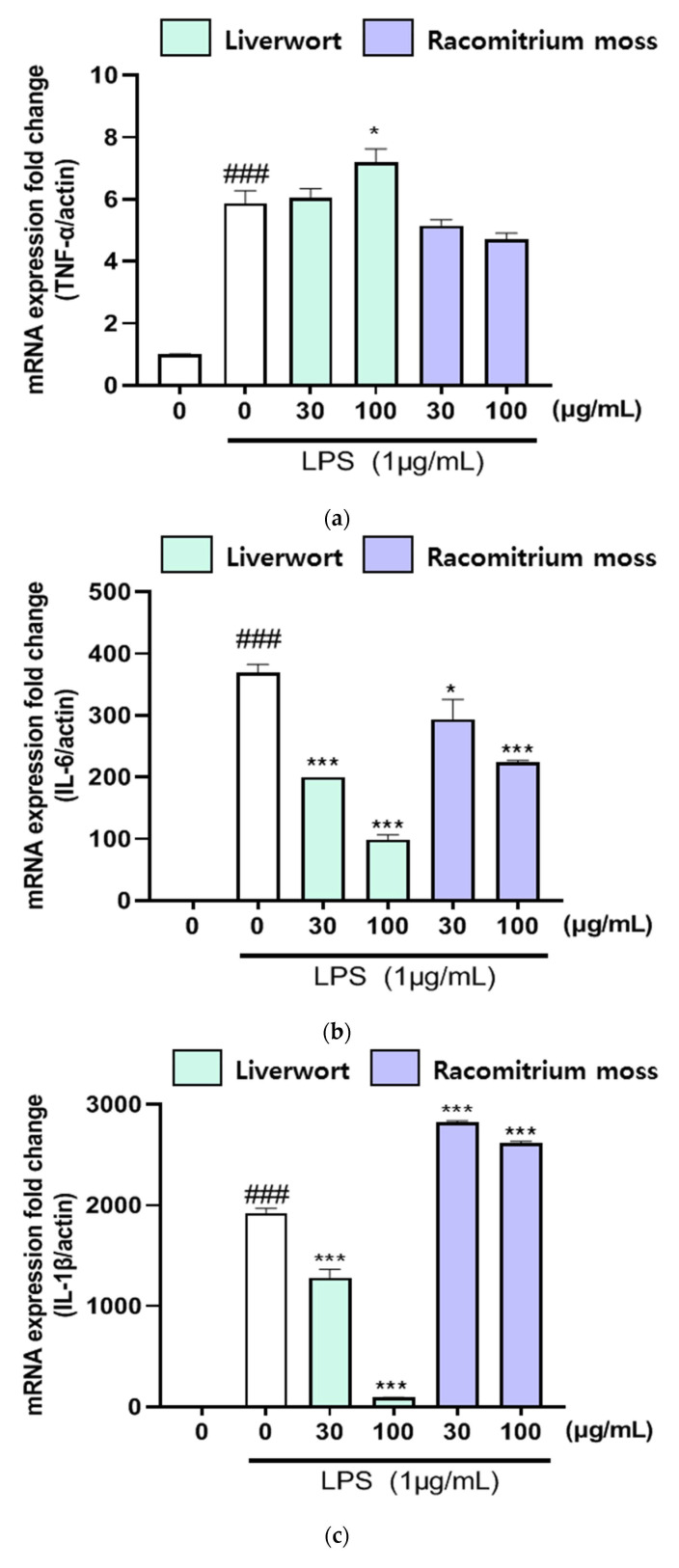
The effect of methanol extract of liverwort and Racomitrium moss on mRNA expressions of TNF-α, IL-6, and IL-1β in LPS-stimulated HaCaT cells. (**a**) mRNA expression of TNF-α; (**b**) mRNA expression of IL-6; (**c**) mRNA expression of IL-1β. Values are mean of three replicate determinations (*n* = 3) ± standard deviation. ### *p* < 0.001 vs. 1% MeOH alone, * *p* < 0.05, *** *p* < 0.001 vs. LPS alone.

We also analyzed the mRNA expression of iNOS, COX-2, TNF-α, IL-6, and IL-1β in LPS-stimulated HaCaT cells using RT-PCR in order to confirm the inhibitory effects of methanol extracts of liverwort and Racomitrium moss on pro-inflammatory mediators. HaCaT cells were pre-treated with two different concentrations of extracts (30 and 100 μg/mL) to determine the mRNA expression of pro-inflammatory mediators stimulated by LPS. Treatment with LPS alone (1 µg/mL) significantly upregulated the mRNA expression of iNOS, COX-2, TNF-α, IL-6, and IL-1β in HaCaT cells (Figure 4 and Figure 5). On the other hand, pre-treatment with methanol extract of liverwort (at 100 μg/mL) significantly (*p* < 0.001) suppressed the mRNA expression of iNOS, COX-2, IL-6, and IL-1β in LPS-stimulated HaCaT cells when compared to that of Racomitrium moss extract. However, there was no significant downregulation of mRNA expression of TNF-α. Further, Racomitrium moss extract did not show significant downregulation of mRNA expression of COX-2. These data suggest that the methanol extract of liverwort and Racomitrium moss effectively reduced the nitrite accumulation by downregulating the mRNA expression of these pro-inflammatory mediators.

The iNOS and COX-2 and their reaction products are highly connected with inflammatory diseases [30]. Previous studies demonstrated that plant extracts/compounds can selectively suppress the expression of iNOS and COX-2. In addition, a strong correlation between NO production and iNOS expression was observed, as shown by other authors [31,32]. The present study also proved that the methanol extract of liverwort remarkably suppressed the expression of iNOS and COX-2 in LPS-stimulated HaCaT cells. Similarly, dollabellane- and ent-kaurane-type diterpenoids isolated from Chinese liverwort, *Lepidozia reptans,* effectively attenuated the mRNA expression of IL-6, IL-β, IL-α, TNF-α, and COX-2 in LPS-stimulated RAW264.7 cells [28].

In LPS-stimulated cell lines, nuclear factor kappa B (NF-κB) is an important transcription factor in the expression of iNOS and COX-2 genes [33,34]. In LPS-induced human keratinocyte HaCaT cells, He et al. [35] found that feruloylserotonin suppressed the toll-like receptor (TLR4)/NF-κB pathway and promoted the translocation of Nuclear factor-erythroid 2 related factor 2 (Nrf2). The chloroform fraction of *Carpinus tschonoskii* inhibited the protein and mRNA of chemokine in HaCaT cells by downregulating STAT1 in the IFN-γ signaling pathway [36]. Another study indicated that peat moss extracts induced the sequestration of NF-κB in the cytoplasm by inhibiting the degradation of IκBα in the LPS-stimulated RAW 264.7 cells. Further, peat moss extracts suppressed the activation of MAPKs and facilitated the activation of Nrf2, and enhanced heme oxygenase-1 (HO-1) expression [30]. A study indicated that miR-127 is involved in the inhibitory effect of Schisandrin A on LPS-induced inflammation injury in HaCaT cells via inactivating p38MAPK/ ERK and JNK signaling pathways [37]. It can be observed that downregulation of the NF-κB and MAPK pathways play a crucial role in the regulation of pro-inflammatory mediators. The suppression of NO production and the downregulation of mRNA expression of various pro-inflammatory mediators are effective therapeutic approaches to block the potentially harmful production of pro-inflammatory mediators by keratinocytes [38]. Moreover, the results of the present study throw some light on the inhibitory effect of liverworts on the production of pro-inflammatory mediators in HaCaT cells.

### 2.4. The Effect of Extracts on the Production of TNF-α, IL-16, and IL-1β

**Figure 6:**
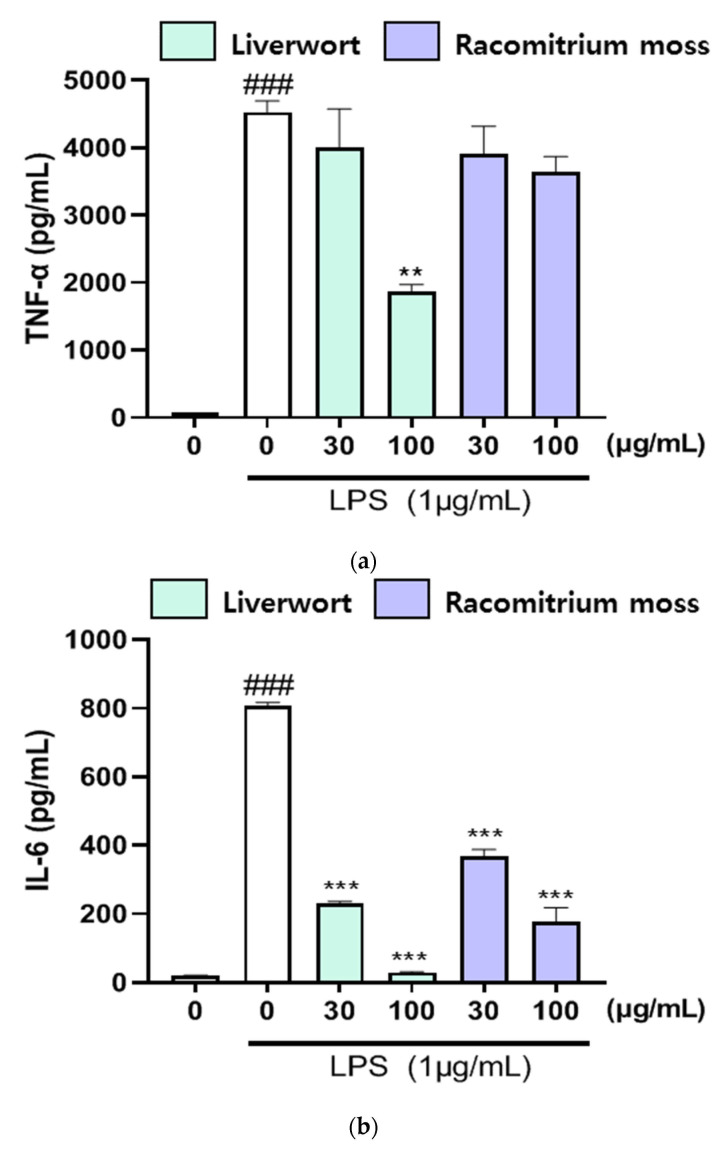

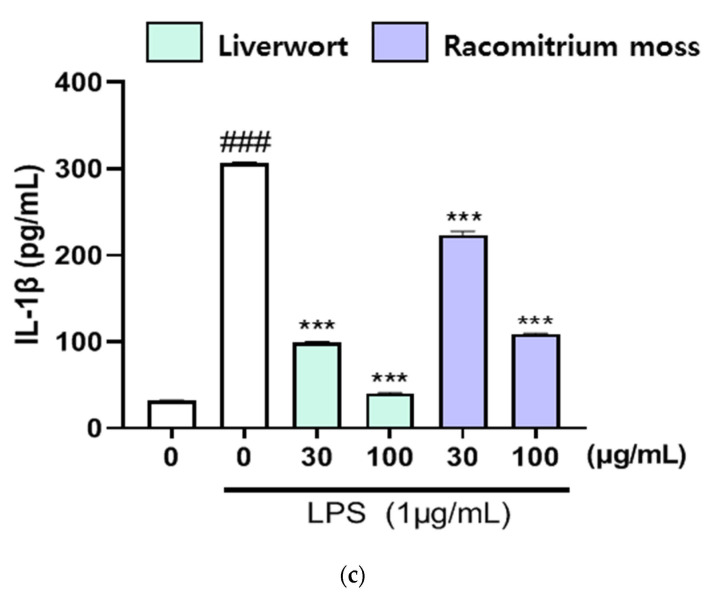
The effect of methanol extract of liverwort and Racomitrium moss on the level of TNF-α, IL-6, and IL-1β in the culture medium of LPS-stimulated HaCaT cells. (**a**) the level of TNF-α; (**b**) the level of IL-6; (**c**) the level of IL-1β. Values are mean of three replicate determinations (*n* = 3) ± standard deviation. ### *p* < 0.001 vs. 1% MeOH alone, ** *p* < 0.01, *** *p* < 0.001 vs. LPS alone.

As shown in Figure 6, the production of TNF-α (4528 pg/mL), IL-6 (808 pg/mL), and IL-1β (306 pg/mL) were enhanced by the LPS treatment. However, LPS-induced production of TNF-α (1867 pg/mL), IL-6 (29 pg/mL), and IL-1β (40 pg/mL) in HaCaT cells were effectively suppressed upon the treatment of methanol extract of liverwort (at 100 μg/mL) than Racomitrium moss extract. Further, Racomitrium moss extract did not show any significant effect on the production of TNF-α. These data indicated that methanol extracts of liverwort and Racomitrium moss could protect HaCaT cells against LPS-induced cell injury.

### 2.5. The Effect of Fractions of Liverwort on Nitric Oxide Production

**Figure 7:**
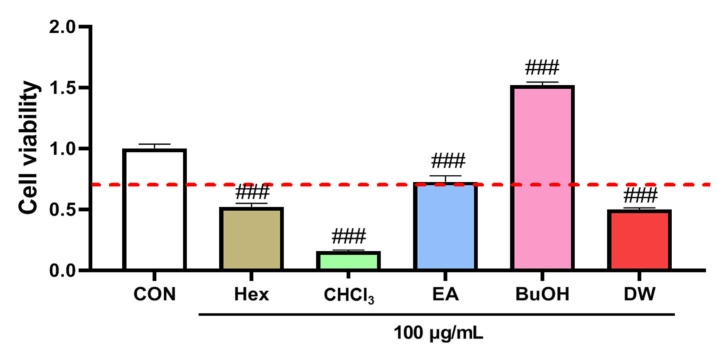
The effect of different fractions (at 100 µg/mL) obtained from methanol extract of liverwort on the cell viability of HaCaT Cells. CON, control; Hex, hexane fraction; CHCl_3_, chloroform fraction; EA, ethyl acetate fraction; BuOH, butanol fraction; DW, water fraction. Values are mean of three replicate determinations (*n* = 3) ± standard deviation. ### *p* < 0.001 vs. 1% MeOH alone.

**Figure 8:**
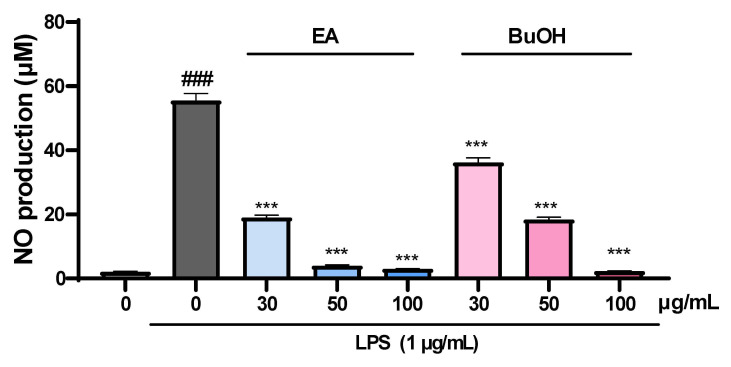
Inhibitory effect of ethyl acetate and butanol fractions of methanol extract of liverwort on NO production by LPS-stimulated HaCaT cells. EA, ethyl acetate fraction; BuOH, butanol fraction; Values are mean of three replicate determinations (*n* = 3) ± standard deviation. ### *p* < 0.001 vs. 1% MeOH alone, *** *p* < 0.001 vs. LPS alone.

In the cell viability assay, butanol fraction obtained from the methanol extract of liverwort exhibited no cytotoxicity effect against HaCaT cells at 100 µg/mL. Moreover, ethyl acetate fraction showed a low cytotoxic effect at 100 µg/mL. However, hexane, chloroform, and water fractions significantly reduced the cell viability (absorbance < 0.7) at the concentration of 100 µg/mL (Figure 7). Hence, ethyl acetate and butanol fractions were selected for further NO production assay. The cells pretreated with ethyl acetate and butanol fractions significantly (*p* < 0.001) inhibited the production of NO in a concentration-dependent manner by reducing the level of nitrite in the medium (Figure 8). Further studies in connection with the isolation of bioactive components from these fractions are under progress.

### 2.6. Liquid Chromatography-Mass Spectrometry (LC-MS) Analysis of Methanol Extracts

**Figure 9:**
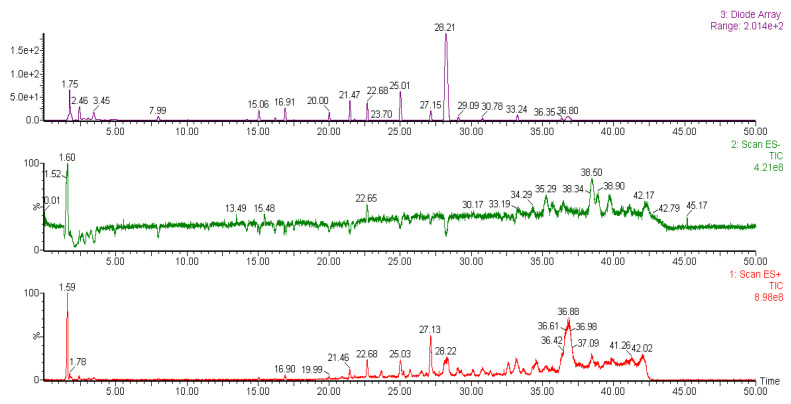
The LC-MS chromatogram of the methanol extract of liverwort.

**Figure 10:**
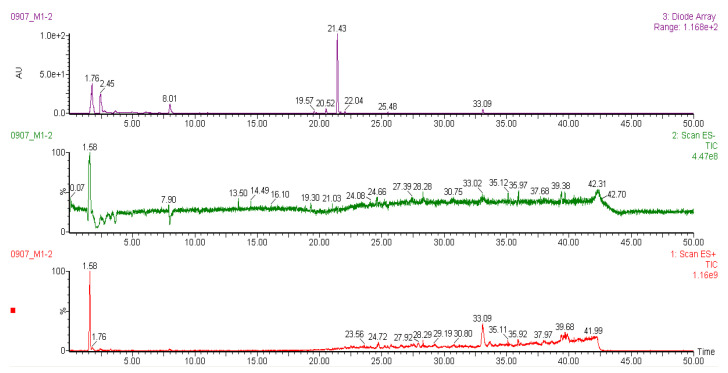
The LC-MS chromatogram of the methanol extract of Racomitrium moss.

**Figure 11:**
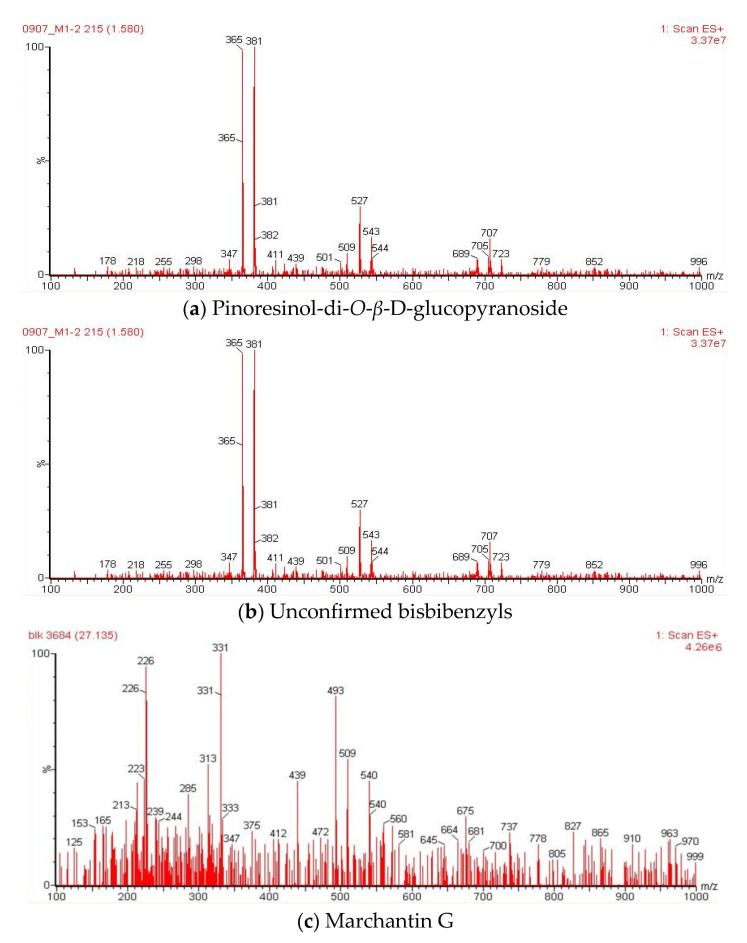
Mass spectra of Pinoresinol-di-*O*-*β*-D-glucopyranoside, unconfirmed bisbibenzyl, and marchantin G from the methanol extract of liverwort.

It was reported that liverworts are an exceptionally rich source of terpenoids, particularly sesqui- and diterpenoids [39]. Tosun et al. [13] also stated that the sesquiterpene-group components are partially responsible for the anti-inflammatory and antinociceptive properties of different liverworts. However, bryophytes contain a variety of other secondary metabolites with potent biological properties. The methanol extracts of liverwort and Racomitrium moss were subjected to LC-MS analysis (Figure 9 and Figure 10). The molecular mass and fragments of the identified compounds in the methanol extracts of liverwort and Racomitrium moss are presented in Table 1. Eleven components were identified from the methanol extract of liverwort and four components were identified from the methanol extract of Racomitrium moss. In positive mode ionization, the mass data show a similar fragmentation profile with *m/z* at 543, 527, and 381, which is correlated to pinoresinol-di-*O*-*β*-D-glucopyranoside (Figure 11) [40]. Pinoresinol-di-*O*-*β*-D-glucopyranoside exhibited remarkable inhibitory activity against the production of PGE_2_ in LPS-induced RAW264.7 cells [41]. In methanol extract of liverwort, peak 9 shows *m/z* at 439, 331, and 226; this compound could be identified as marchantin G [42]. The peak 3 at *m/z* at 423, 411, and 213 and the peak 8 *m/z* at 479, 239, and 211 represent unidentified bisbibenzyls (Figure 11) [43,44]. In addition, peak 2 from Racomitrium moss shows *m/z* at 931, 767, and 753 corresponding to dioscoreside A [45].

The LC-MS data indicated that the methanol extract of liverwort mainly contains the bisbibenzyl group of components. They are aromatic compounds and have one or two diaryl ether or biphenyl bonds mainly found in liverworts, including *Riccardia*, *Marchantia*, *Plagiochila*, etc. [46]. Previously, seven confirmed bisbibenzyls and twelve unconfirmed bisbibenzyl components were detected in the ethanol extract of *M. polymorpha* [44]. Sabovljević et al. [42] studied the comparison of LC-MS analysis of methanol extracts from natural and cultured liverwort. The results indicated the presence of marchantin A in both natural and cultured liverwort. On the other hand, marchantin E, G, and/or C, and dehydromarchantin A were found only in the cultured liverwort. Harinantenaina et al. [46] investigated the inhibitory effect of nineteen bisbibenzyls on NO production in LPS-stimulated RAW 264.7 cells. Among the tested components, marchantin A exhibited strong inhibitory activity against NO production and mRNA expression of iNOS. Previous studies demonstrate that the presence of bisbibenzyl-type components in the methanol extract of liverwort may be responsible for its anti-inflammatory activity.

## 3. Materials and Methods

### 3.1. Sample Collection

*M. polymorpha* and *R. canescens* samples were collected from Songjung-ri, Jinbu-Myeon, Pyeongchang, in Korea during October 2020 (Figure 1). Collected samples were kept in a 4 °C freezer and transported to the laboratory. The soil and plant debris in the samples was removed by washing in running tap water. The samples were authenticated and deposited in the Herbarium, National Institute of Biological Resources (NIBR), Korea, with voucher numbers NIBRMS 0000107499 and NIBRMS 0000107500, respectively.

### 3.2. Preparation of Methanol Extract

Plant samples were dried at room temperature, pulverized using a grinder (HANIL HMF-3260S, Hanil Electric Co., Seoul, Korea) up to 0.6 mm. One kilogram of powdered leaves was extracted twice with 4-L of methanol per extraction for 2 days and filtered. The combined filtrates were concentrated using a rotary vacuum evaporator at 40 °C (EYELA NE-1101, Tokyo Rikakikai Co., Ltd., Tokyo, Japan) and the concentrate was dissolved with 50 mL of distilled water. Then the extract was dried using a freeze dryer (FD5505, ILSHIN BIOBASE, Dongduchon, Korea). The obtained extracts of liverwort and Racomitrium moss were dissolved and diluted to 10 mg/mL in methanol as a stock solution.

### 3.3. Cell Culture

HaCaT cells (human epidermal fibroblast) were provided by the Food Chemistry laboratory at Kangwon National University (Prof. Lee). Cell culture medium was used in Dulbecco’s Modified Eagle’s Medium (DMEM) with 100 units/milliliter penicillin-streptomycin (P/S) and 10% fetal bovine serum (FBS) [47]. Thereafter, the cells were cultured at 37 °C and 5% CO2, followed by subculture every three days, respectively.

### 3.4. Cell Viability Analysis

Cell viability was estimated for cytotoxicity of methanol extract of liverwort and Racomitrium moss using the MTT assay. Cultured cells were treated with methanol extract of liverwort and Racomitrium moss at 12.5–100 μg/mL for 24 h. After incubation with MTT solution diluted 10:1 (5 mg/mL in PBS) at 37 °C for 4 h, purple formazan was formed in the cells. The solution in each well was completely removed and then the purple formazan crystals were dissolved in DMSO and isopropyl alcohol at 1:1 (100 μL/well). The optical density was measured at 540 nm using a SpectraMax 190 Microplate Reader (Molecular Devices, San Jose, CA, USA).

### 3.5. Measurement of Nitric Oxide

HaCaT cells were pre-treated with methanol extract of liverwort and Racomitrium moss at 30 and 100 μg/mL for 1 h, followed by stimulation with LPS (1 μg/mL) for 24 h. Nitrite accumulation in the culture medium as an indicator of NO production was measured using Griess reagent [48]. The culture supernatant (100 μL) was mixed with 100 μL of Griess reagent (equal volumes of 1% (*w*/*v*) sulfanilamide in 0.1% (*w*/*v*) naphthyl ethylenediamine-HCl and 5% (*v*/*v*) phosphoric acid) for 10 min [47]. The optical density was measured at 540 nm using a SpectraMax 190 Microplate Reader (Molecular Devices, San Jose, CA, USA). The amount of nitrite in the medium was determined with reference to a sodium nitrite (NaNO_2_) standard curve.

### 3.6. RNA Isolation and Real Time-Polymerase Chain Reaction (RT-PCR)

RT-PCR was used to estimate the mRNA expression of iNOS, COX-2, TNF-α, IL-6, and IL-1β. Total RNA was extracted from HaCaT cells using RNAiso PLUS. Total RNA (1 μg) was used to generate cDNA by reverse transcription using All-in-One First-Strand cDNA Synthesis SuperMix [49]. The synthesized cDNA was used as a template for qRT-PCR using QuantStudio 3 (Applied Biosystems, Foster City, CA, USA) system with FG POWER SYBR Green PCR master mix and gene-specific primers (Table 2) [47]. A dissociation curve analysis of iNOS, COX-2, TNF-α, IL-6, IL-1β, and β-actin showed a single peak. Expression levels of target genes were quantified from duplicate measurements and normalized with the 2^−ΔΔCT^ method relative to β-actin.

### 3.7. Enzyme-Linked Immunosorbent Assay (ELISA)

HaCaT cells were pre-treated with methanol extract of liverwort and Racomitrium moss at 30 and 100 μg/mL for 4 h and then treated with LPS (1 μg/mL) for an additional 20 h. The supernatants were collected and analyzed for the levels of TNF-α, IL-6, and IL-1β (Invitrogen, Carlsbad, CA, USA) using ELISA kits according to the manufacturer’s protocol.

### 3.8. Fractionation of Methanol Extract of Liver Wort and Nitric Oxide Measurement

The methanol extract of liverwort was prepared as mentioned in Section 2.2. The crude methanol extract was suspended in deionized water and the aqueous solution was sequentially partitioned with hexane, chloroform (CHCl_3_), ethyl acetate (EA), butanol (BuOH). The obtained fractions, in addition to the aqueous solution, that remained after the extraction were filtered and concentrated and dried under vacuum. The crude fractions were used for the assessment of cell viability and nitric oxide production assay using HaCaT cells.

### 3.9. Liquid Chromatography-Mass Spectrometry (LCMS) Analysis of Methanol Extracts

The chemical profile of methanol extracts from liverwort and Racomitrium moss was identified by LC-MS method using the instrument Waters auto-purification system with Waters 3100 single mass system (Waters, USA). The LC system was connected to 3100 single mass (100–1000 *m*/*z*) and 2998 Photodiode Array Detector (230–600 nm). Analyte separation was performed on a SunFire C18 (150 mm × 4.6 mm × 5 mm) with a gradient mobile phase consisting of 0.1% trifluoroacetic acid in water (solvent A) and acetonitrile (solvent B). The compositions of mobile phases consisted of the following multistep linear gradient: 0–10 min, 10% B and 90% A; 10–20 min, 20% B and 80% A; 20–30 min, 30% B and 70% A; 30–40 min, 50 % B and 50% A; 40–50 min, 70% B and 30% A. The flow rate was set at 1 mL/min. The sample injection volume was 10 µL. All chromatographic procedures were performed at ambient temperature and the corresponding peaks from the LC-MS analysis of all the samples were identified by comparison with the literature.

### 3.10. Statistical Analysis

All data analyses were done using GraphPad Prism Version 8.0 (GraphPad, La Jolla, CA, USA). The values expressed were means of three replicate determinations ± SD. All results were analyzed using the Student–Newman–Keuls test for multiple comparisons after analyzed with a one-way analysis of variance (ANOVA). Statistical significance was set at *p* < 0.05.

## 4. Conclusions

The data of the present study demonstrate that the methanol extract of liverwort effectively inhibited the mRNA expression (except TNF-α) and production of pro-inflammatory mediators in LPS-stimulated HaCaT cells when compared with Racomitrium moss extract. It could be concluded that the methanol extract of liverwort is a potential candidate for the development of an anti-inflammatory drug against inflammation-mediated skin diseases. Further isolation of biologically active metabolites from ethyl acetate and butanol fractions and elucidation of their anti-inflammatory mechanism(s) are required to facilitate the development of therapeutic agents for inflammation-mediated skin diseases.

## Figures and Tables

**Table 1:** Identification of chemical constituents from methanol extracts of liverwort and Racomitrium moss by LC-MS.

Peak	Tentative Identification	Retention Time (min)	Molecular Mass (Da)	Fragments (m/z)	References
**Liverwort**					
1	Pinoresinol-di-*O*-*β*-D-glucopyranoside	1.59	705	707, 543, 527, 381, 365	[40]
2	Unknown	1.78	517	826, 625, 517, 381, 204	-
3	Unconfirmed bisbibenzyl	15.05	421	463, 423, 411, 309, 213	[43]
4	Unknown	16.90	447	621, 447, 271, 145	-
5	Unknown	19.90	674	970, 806, 772, 674, 597	-
6	Unknown	21.46	565	903, 858, 565, 431, 322,	-
7	Unknown	22.68	658	917, 833, 658, 483, 158	-
8	Unconfirmed bisbibenzyl	25.03	424	479, 452, 417, 239, 142	[44]
9	Marchantin G	27.13	454	493, 439, 331, 313, 226	[42]
10	Unconfirmed bisbibenzyl	28.22	454	463, 458, 333, 224, 197	[44]
11	Unknown	36.88	599	975, 907, 599, 256, 157	-
**Racomitrium moss**					
1	Pinoresinol-di-*O*-*β*-D-glucopyranoside	1.58	705	707, 543, 527, 381, 365	[40]
2	Dioscoreside A	7.94	784	931, 793, 767, 753, 268	[45]
3	Unknown	25.75	748	748, 657, 603, 505, 411	-
4	Unknown	33.09	518	518, 497, 263, 215, 205	-

**Table 2:** Primer sequences used for quantitative real-time PCR analysis.

Target Gene		Primer Sequence
iNOS	Forward	5′-CATTGATCTCCGTGACAGCC-3′
	Reverse	5′-CATGCTACTGGAGGTGGGTG-3′
COX-2	Forward	5′-GCAGCCATTTCCTTCTCTCC-3′
	Reverse	5′-TGCTGTACAAGCAGTGGCAA-3′
TNF-α	Forward	5′-CTG ATG AGA GGG AGG CCA TT-3′
	Reverse	5′- AGC ACA GAA AGC ATG ATC CG-3′
IL-6	Forward	5′-AAGTGCATCATCGTTGTTCATACA-3′
	Reverse	5′-GAGGATACCACTCCCAACAGACC-3′
IL-1β	Forward	5′-TCGTTGCTTGGTTCTCCTTG-3′
	Reverse	5′-ACCTGCTGGTGTGTGACGTT-3′
β-actin	Forward	5′-TCAGCAATGCCTGGGTACAT-3′
	Reverse	5′-ATCACTATTGGCAACGAGCG-3′

## Data Availability

The data presented in this study are available within the article.
